# Effects of different force directions of intra-oral skeletally anchored maxillary protraction on craniomaxillofacial complex, in Class III malocclusion: a 3D finite element analysis

**DOI:** 10.1590/2177-6709.27.5.e2220377.oar

**Published:** 2023-01-06

**Authors:** Dhiraj GARG, Priyank RAI, Tulika TRIPATHI, Anup KANASE

**Affiliations:** 1 Maulana Azad Institute of Dental Sciences, Department of Orthodontics and Dentofacial Orthopaedics (New Delhi, India).

**Keywords:** Finite element analysis, Skeletal Class III treatment, Miniplate

## Abstract

**Introduction:**

The intra-oral skeletally anchored maxillary protraction (I-SAMP) has been found to be an effective treatment for skeletal Class III malocclusion.

**Objective:**

This *in-silico* study explored the influence of different force directions of intra-oral skeletally anchored Class III elastics on the changes in craniomaxillofacial complex, using finite element analysis.

**Methods:**

A 3-dimensional (3D) finite element model of the craniomaxillofacial bones including circummaxillary sutures was constructed with high biological resemblance. A 3D assembly of four miniplates was designed and fixed on the maxilla and mandible of the finite element model. The model was applied with 250g/force at the miniplates at three angulations (10°, 20°, and 30°) from the occlusal plane, to measure stress and displacement by using the ANSYS software.

**Results:**

The zygomaticotemporal, zygomaticomaxillary, and sphenozygomatic sutures played significant roles in the forward displacement and counterclockwise rotation of maxilla and zygoma, irrespective of the angulation of load application. The displacements and rotations of the zygomatico-maxillary complex decreased gradually with an increase in the angle of load application between miniplates from 10° to 30°. The mandible showed negligible displacement, with clockwise rotation.

**Conclusions:**

The treatment effects of I-SAMP were corroborated, with insight of displacement patterns and sutures involved, which were lacking in the previously conducted 2D and 3D imaging studies. The prescribed angulation of skeletally anchored Class III elastics should be as low as possible, since the displacement of zygomatico-maxillary complex increases with the decrease in angulation of the elastics.

## RESUMO

**Introdução:**

A protração maxilar com ancoragem esquelética intrabucal (I-SAMP) tem sido considerada um tratamento efetivo para a má oclusão esquelética de Classe III.

**Objetivo:**

O presente estudo *in silico* avaliou, usando análise de elementos finitos, a influência de diferentes direções da força dos elásticos Classe III com ancoragem esquelética intrabucal nas mudanças no complexo craniomaxilofacial.

**Métodos:**

Um modelo de elementos finitos tridimensional (3D) dos ossos craniomaxilofaciais, incluindo as suturas circum-maxilares, foi construído, com alta semelhança biológica. Uma montagem 3D de quatro miniplacas foi projetada e fixada na maxila e na mandíbula do modelo de elementos finitos. O modelo foi aplicado com o uso de 250g/força nas miniplacas em três angulações (10°, 20° e 30°) em relação ao plano oclusal, para medir as tensões e os deslocamentos, usando o programa ANSYS.

**Resultados:**

As suturas zigomaticotemporal, zigomaticomaxilar e esfenozigomática desempenharam um papel significativo no deslocamento para anterior e na rotação anti-horária da maxila e do zigoma, independentemente da angulação na aplicação da força. Os deslocamentos e as rotações do complexo zigomático-maxilar diminuíram gradualmente com o aumento de 10° para 30° no ângulo de aplicação da força entre as miniplacas. A mandíbula apresentou deslocamento irrelevante, com rotação no sentido horário.

**Conclusões:**

Os efeitos do tratamento com I-SAMP foram corroborados, com um vislumbre dos padrões de deslocamento e das suturas envolvidas, que não existiam nos estudos com imagens 2D e 3D realizados anteriormente. A angulação dos elásticos Classe III ancorados esqueleticamente deve ser a menor possível, visto que o deslocamento do complexo zigomático-maxilar aumenta com a redução no ângulo dos elásticos.

## INTRODUCTION

The use of skeletally anchored maxillary protraction (SAMP) for orthopedic treatment of maxillary retrognathia has reduced the dentoalveolar and skeletal side effects of tooth-borne devices and enhanced maxillary protraction.^1^ ^-^ ^6^ The use of dentally anchored maxillary protraction (DAMP) with rapid maxillary expansion (RME) and facemask is currently limited to the deciduous or early mixed dentitions.^3^ However, Intra-oral Skeletally Anchored Maxillary Protraction (I-SAMP) with Class III elastics can be successfully used in the late mixed or permanent dentition phases.^4^ ^,^ ^5^

By using intermaxillary elastics between miniplates on zygomatic crests of the maxilla and in the anterior mandibular region, De Clerck et al^4^ introduced a new perspective to the orthopedic treatment of Class III malocclusions. With this method, intermaxillary traction can be applied almost 24 hours a day.^5^ Cevidanes et al^6^ found that I-SAMP had two to three millimeters more maxillary advancement than RME combined with facemask.

Originally developed by Courant, finite element method (FEM) is a computer-aided numerical technique that has been used to study the nature of stress and strain induced by orthodontic forces during tooth movement.^7^ Based on the division of a complex structure into smaller sections called elements, FEM provides detailed information on the physiological reactions in tissues after applying orthopedic forces.^8^ A FEM study in which protraction forces were applied only on maxilla at different location and directions concluded that by varying the mechanics directions, different magnitudes of forward, downward, and rotational movements of the maxilla can be achieved.^9^

Although I-SAMP is an established technique for correction of skeletal Class III, currently there is a paucity of data on the optimum load and appropriate direction of Class III elastics for this type of orthopedic traction. Further, there is a need for a collective finite element study to evaluate the stress pattern and displacement in different craniofacial sutures and diverse bones of craniomaxillofacial complex, and to improve the mechanics and results of this treatment modality. Thus, the aim of this study was to explore the influence of different force directions of I-SAMP on craniomaxillofacial complex, by using the finite element analysis.

## MATERIAL AND METHODS

### A) CONE BEAM COMPUTED TOMOGRAPHY RAW DATA

Cone beam computed tomography (CBCT) scan (i-CAT Vision; Hatfield, PA), (120kVp, 5mA, 130.0 FOV, 4sec) of craniomaxillofacial complex of a 10 year old female with Class III malocclusion who had a retrusive maxilla with protrusive mandible and an anterior crossbite was taken from patient’s diagnostic data records. Experimental protocols were reviewed and approved by Institutional Ethical Committee (MAIDS/EC/2016/10/04).

### B) FINITE ELEMENT ANALYSIS

#### Pre-processing

**Figure 1:**
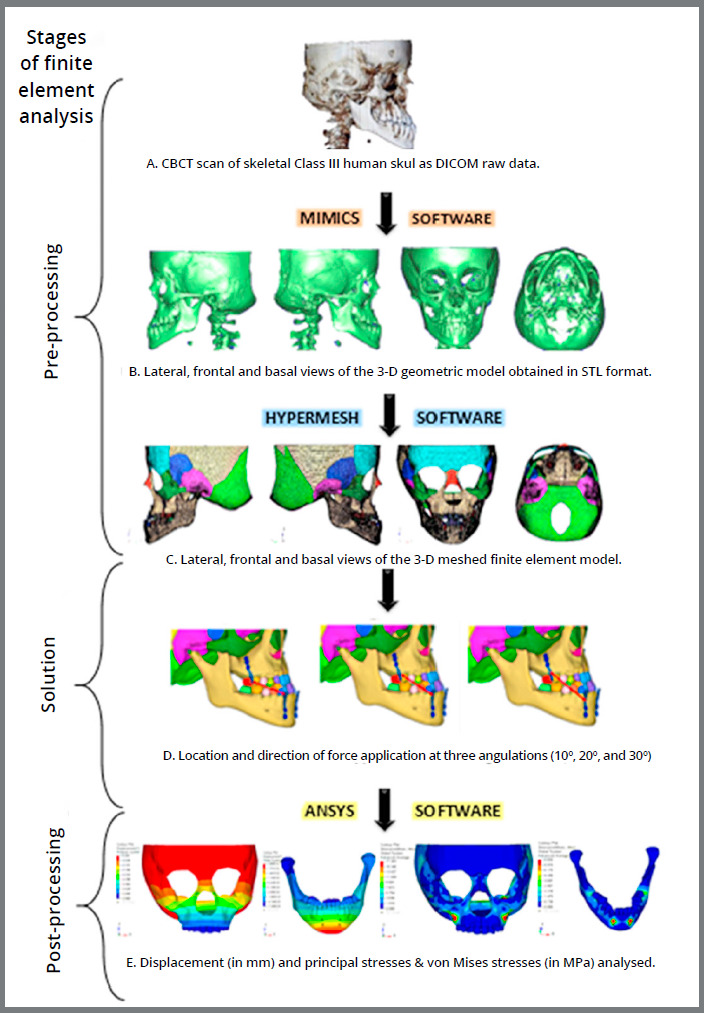
Flowchart of experimental design.

**Figure 2:**
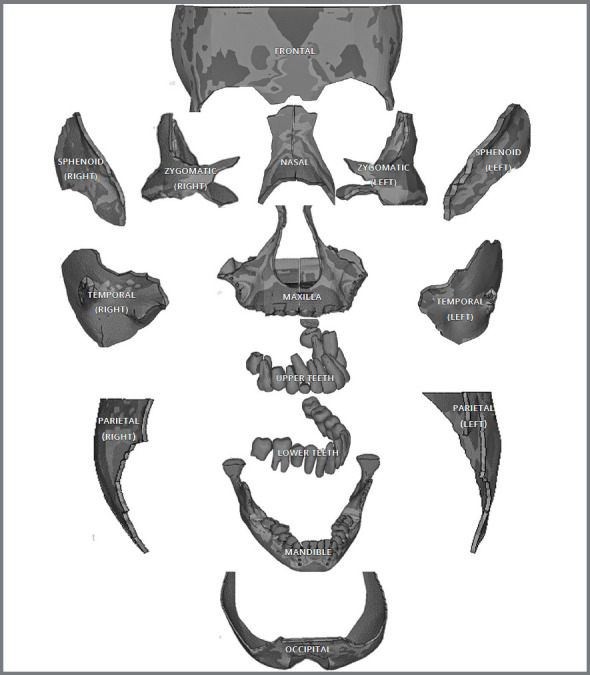
3D segmented different craniofacial bones and teeth.

Raw volumetric reconstructive data from the CBCT scan (Fig 1A) was saved as digital imaging and communications in medicine (DICOM) files, and then imported into finite element modeling software (Mimics 8.11, Materialise: Leuven, Belgium), and a 3D model of the patient’s skull was reconstructed (Fig 1B). The 3D model of the skull was then segmented into separate 3D models of sutures, craniofacial bones, and teeth, to refine these structures (Fig 2).

Nine craniofacial sutural systems (frontonasal, frontomaxillary, zygomaticomaxillary, zygomaticotemporal, zygomaticofrontal, pterygomaxillary, internasal, nasomaxillary, and sphenozygomatic) were assimilated into the model, with an even suture thickness of 0.8 mm. This geometric model in stereolithography (STL) file format was imported into the Hypermesh version 13.0 software (Altair Computing, Inc, Troy, MI), which was used to generate a volume mesh and assign the material properties according to the final geometric model. The model was meshed into 465,091 tetrahedral elements and 101,247 nodes. The combined 3D mesh model of craniofacial bones and sutures is shown in Figure 1C.

A 3D finite element model of miniplates - with specifications like holes (3 - upper miniplate / 2 - lower miniplate); hooks (3); thickness (0.80mm); length (upper =31.65mm & lower =21mm); hole diameter (2mm); distance between the centers of the holes (5.50mm) (S. K. Surgicals, Maharashtra, India) - was designed. It was based on 3D computer-aided design data, and fixed bilaterally according to the anatomic shape on the infrazygomatic crest of the maxilla and in between canine and lateral incisor of the mandible, by the projection method. The materials used in the discretized model were assumed to be isotropic, homogeneous and linearly elastic ,and the mechanical properties were assigned to the model as depicted in Table 1.^10^

**Table 1:** Young’s modulus and Poisson’s ratio for the materials used in this study.

Material	Young’s modulus (MPa)	Poisson’s ratio
Cortical bone	1.37 x 10^4^	0.30
Cancellous bone	7.9 x 10^3^	0.30
Miniplate	1.05 x 10^5^	0.33
Miniscrew	1.05 x 10^5^	0.33
Suture	7	0.40
Tooth	2.07 x 10^4^	0.30
Periodontal ligament	50.00	0.49

#### Solution

**Figure 3:**
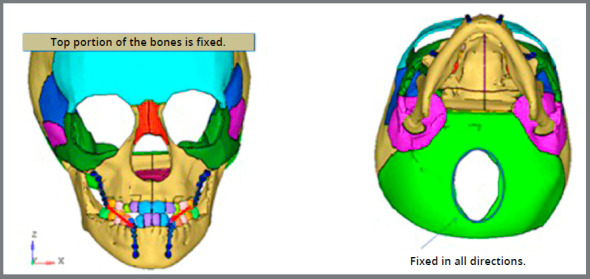
Boundary conditions on the finite element analysis.

**Figure 4:**
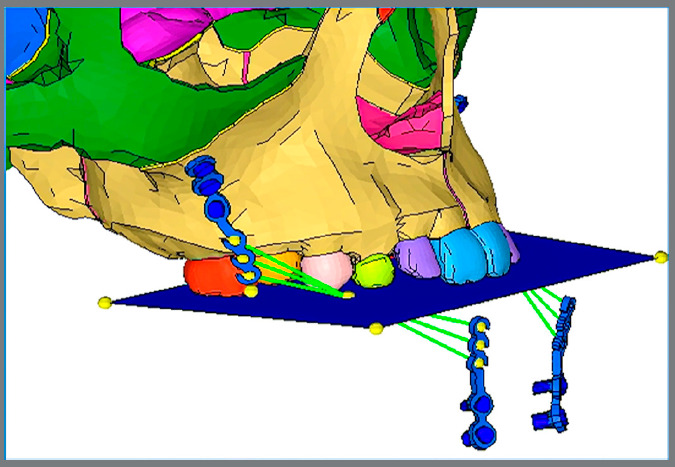
Illustration showing three combinations of force application between different hooks on the distal ends of both maxillary and mandibular miniplates.

Nodes along the foramen magnum of occipital bone and all nodes of the cranium lying on a symmetrical plane bypassing frontal, parietal and occipital bones were fully constrained by all degrees of freedom, zero displacement and zero rotation (Fig 3).^10^ Protraction forces (250g/f side) were applied in between different hooks of both maxillary and mandibular miniplates bilaterally (Fig 4).^4^ ^,^ ^5^

**Figure 5:**
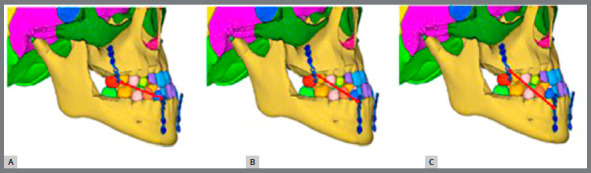
Three analytic models based on locations and angulations of the force: A) 10° angulation; B) 20° angulation; C) 30° angulation.

Three analytic models (Fig 5) were developed based on locations and angulations of the force application as in 10° angulation model, 250g of load connected between distal hooks of upper and lower miniplates at 10° to occlusal plane. Similarly, in 20° angulation model, load was connected from central hook to central hook at 20°; and in 30° angulation model, from mesial hook to mesial hook at 30°.

#### Post-processing

The database file from the HYPERMESH software was transferred to the ANSYS v. 12.1 simulation software (ANSYS Inc, Canonsburg, Pa). Utilizing the ANSYS software, areas of high stress distribution in the nine circummaxillary sutures as well as in craniomaxillofacial bones and displacement of the surface landmarks in the maxillofacial bones were identified and analyzed. Three principal and von Mises stresses and displacement in Y, and Z-axes were output through stress and displacement nephrogram, respectively. 

Mathematically, von Mises stress, σ_v_, is expressed in three dimensions as:

$$\sigma_{v}=\sqrt{\frac{{\left(\sigma_{1}-\sigma_{2}\right)}^{2}+{\left(\sigma_{2}-\sigma_{3}\right)}^{2}+{\left(\sigma_{3}-\sigma_{1}\right)}^{2}}{2}}$$

where σ_1_, σ_2_, and σ_3_ are the principal stresses. 

In addition to bounding the principal stresses to prevent ductile failure, the Von Mises stress also gives a reasonable estimate of fatigue failure, especially for repeated tensile and tensile-shear loading. Thus, finite element analysis results are typically presented as Von Mises stress. The principal stresses are the maximum and minimum normal stresses in a plane, always perpendicular to each other, and oriented in directions for which the shear stresses are zero.

The pattern of stress distribution differed along the various sutures in all three dimensions. For comparison, maximum values of first, second, and third principal stresses, and von Mises stresses were considered, respectively. Positive value of principal stresses depicted tensile stress, while negative value indicated compressive stress along the sutures.

To measure the amount of displacement of the maxillofacial complex, nodes on the skull were selected and the displacement values were exported to Microsoft Excel for analysis and graphing. The displacement in the transverse plane was not analyzed, since the applied force was symmetrical in this plane. The forward direction of Y-axis indicates a negative value, and the backward direction of Y-axis indicates a positive value; the upward direction of Z-axis indicates a positive value, and the downward direction of Z-axis indicates a negative value.

### SUPERIMPOSITION

The ANSYS software generated the superimposed contours of displacements automatically when the superimposition button was chosen. The undeformed model (without applied force) was at the bottom, and the deformed model (with applied force) was on top of it, according to the 3-dimensional coordinates. All maxillofacial bones were a best-fit superimposition, since a 3-dimensional finite element model was generated under a 3-dimensional coordinate system. To make the deformation of the 3-dimensional models seen directly, the same local 3-dimensional coordinate system and amplification coefficient were set.

## RESULTS

### STRESS DISTRIBUTION AMONG THE MAXILLOFACIAL BONES

**Figure 6:**
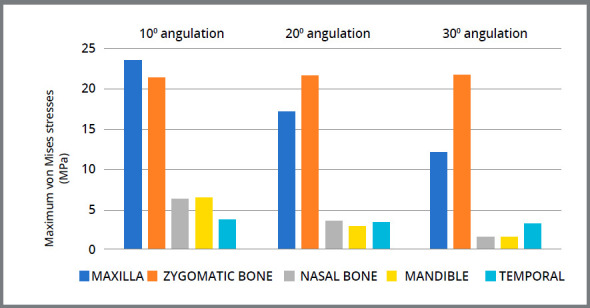
Maximum von Mises stresses in the maxillofacial bones.

The magnitude of maximum von Mises stresses at three different angulations varied from 1.5 to 23.7 megaPascals, which in most of the bones decreases when the angulation of force is increasing, as depicted in Figure 6. Among all the maxillofacial bones, maxilla and zygomatic bone showed highest stresses. In the maxilla, maximum stresses were seen around the site of fixation of miniplates; while second highest von Mises stresses was perceived in zygomatic bone in the lower region of temporal process. In mandible, higher stresses were seen only at the site of miniplates fixation, ranging from 1.6 to 6.6 MPa.

### STRESS DISTRIBUTION ALONG THE SUTURES

**Figure 7:**
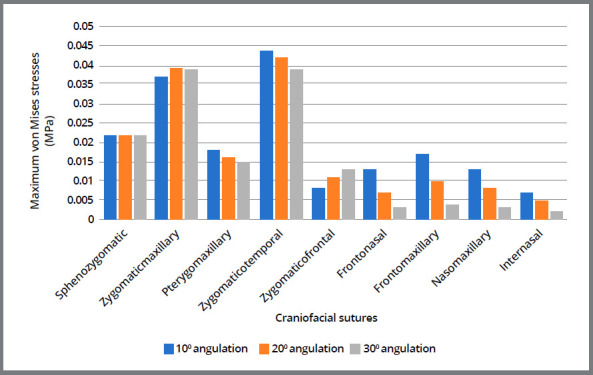
Maximum von Mises stresses pattern along the craniofacial sutures.

Stress pattern with force application at 10°, 20°, and 30° to occlusal plane is tabulated in Tables 2, 3 and 4. The highest amount of von Mises stresses was seen with respect to the zygomaticotemporal sutures (ranging from 0.044MPa to 0.039MPa), as depicted in Figure 7. Second highest stress concentration was seen with the zygomaticomaxillary sutures, followed by the sphenozygomatic sutures. All the three principal stresses were tensile in nature in zygomaticotemporal and pterygomaxillary sutures, while compressive nature was seen with frontomaxillary, frontonasal, and internasal sutures.

**Table 2:** Stress pattern along craniofacial sutures when force applied at 10° to occlusal plane (in Megapascal).

	S1	S2	S3	SEQV
Sphenozygomatic suture	0.035	0.019	-0.016	0.022
Zygomaticomaxillary suture	0.060	0.033	-0.037	0.037
Pterygomaxillary suture	0.037	0.021	0.017	0.018
Zygomaticotemporal suture	0.099	0.065	0.064	0.044
Zygomaticofrontal suture	0.023	0.015	-0.023	0.008
Frontonasal suture	-0.027	-0.031	-0.039	0.013
Frontomaxillary suture	-0.023	-0.023	-0.037	0.017
Nasomaxillary suture	0.017	-0.016	-0.026	0.013
Internasal suture	-0.014	-0.014	-0.021	0.007

S1, S2, S3, and SEQV = Maximum first, second, and third principal stresses, and von Mises stress, respectively.

**Table 3:** Stress pattern along craniofacial sutures when force applied at 20° to occlusal plane (in Megapascal).

	S1	S2	S3	SEQV
Sphenozygomatic suture	0.038	0.021	-0.016	0.022
Zygomaticomaxillary suture	0.064	0.035	-0.038	0.039
Pterygomaxillary suture	0.033	0.02	0.016	0.016
Zygomaticotemporal suture	0.093	0.061	0.061	0.042
Zygomaticofrontal suture	0.027	0.017	0.016	0.011
Frontonasal suture	-0.016	-0.019	-0.024	0.007
Frontomaxillary suture	-0.013	-0.013	-0.021	0.010
Nasomaxillary suture	-0.008	-0.009	-0.014	0.008
Internasal suture	-0.009	-0.009	-0.014	0.005

S1, S2, S3, and SEQV = Maximum first, second, and third principal stresses, and von Mises stress, respectively.

**Table 4:** Stress pattern along craniofacial sutures when force applied at 30° to occlusal plane (in Megapascal).

	S1	S2	S3	SEQV
Sphenozygomatic suture	0.046	0.028	0.024	0.022
Zygomaticomaxillary suture	0.065	0.036	-0.033	0.039
Pterygomaxillary suture	0.030	0.017	0.014	0.015
Zygomaticotemporal suture	0.085	0.056	0.056	0.039
Zygomaticofrontal suture	0.032	0.020	0.018	0.013
Frontonasal suture	-0.006	-0.007	-0.009	0.003
Frontomaxillary suture	-0.004	-0.005	-0.006	0.004
Nasomaxillary suture	0.003	-0.002	-0.004	0.003
Internasal suture	-0.004	-0.005	-0.007	0.002

S1, S2, S3, and SEQV = Maximum first, second, and third principal stresses, and von Mises stress, respectively.

### DISPLACEMENT PATTERN OF THE MAXILLOFACIAL BONES

**Figure 8:**
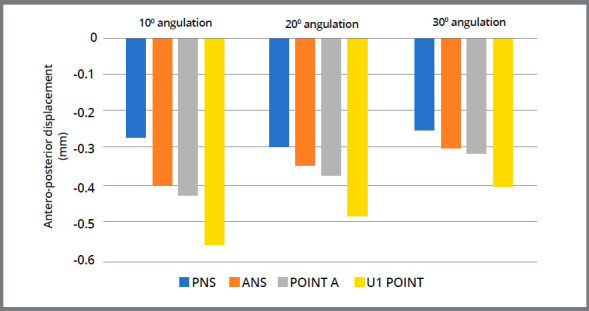
Antero-posterior displacement of the surface landmarks of maxilla.

**Figure 9:**
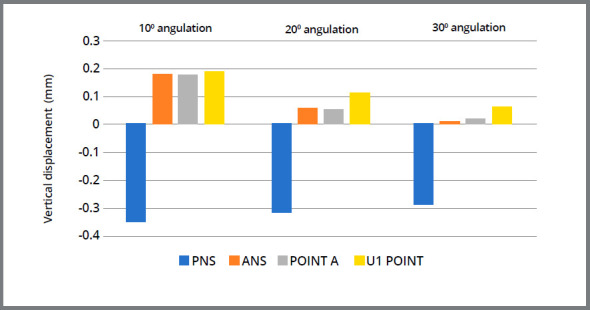
Vertical displacement of the surface landmarks of maxilla.

The amount of displacement at each surface landmark was compared separately on the Y and Z-axes, as tabulated in Table 5. The graphic representations of displacement (in mm) of surface landmarks of maxilla are shown in Figures 8 and 9. FEM analysis showed that the entire maxilla moved forward, which was evident with the forward movement of anterior nasal spine (ANS), Point A, U1 point and body of the zygomatic bone. Similarly, the maxilla also showed rotation in anti-clockwise direction.

**Table 5:** Displacement of the surface landmarks in the maxillofacial structures when 250g force was applied (mm).

		Angle of force 10° to occlusion		Angle of force 20° to occlusion		Angle of force 30° to occlusion	
		Y (mm)	Z (mm)	Y (mm)	Z (mm)	Y (mm)	Z (mm)
Maxilla	Frontal process	0.023	0.122	0.005	0.102	-0.011	0.032
	ANS	-0.396	0.180	-0.347	0.055	-0.296	0.010
	Point A	-0.429	0.180	-0.371	0.012	-0.313	0.020
	U1 point	-0.560	0.196	-0.483	0.115	-0.405	0.065
	PNS	-0.336	-0.344	-0.296	-0.321	-0.253	-0.295
Mandible	L1 point	-0.007	-0.032	-0.003	-0.018	-0.003	-0.016
	Point B	0.008	-0.032	0.005	-0.018	0.005	-0.016
	Pogonion	0.017	-0.032	0.010	-0.018	0.009	-0.016
	Gonion	-0.001	0.000	0.000	-0.001	0.000	-0.001
	Condylion	-0.007	0.004	-0.003	0.002	-0.003	0.001
Zygomatic bone	Body	-0.164	-0.025	-0.120	-0.044	-0.113	-0.055
	Frontal process	0.038	0.022	0.016	-0.013	0.006	-0.002
	Maxillary process	-0.326	0.069	-0.228	0.017	-0.208	-0.055
	Temporal process	-0.124	-0.119	-0.092	-0.104	-0.089	-0.109
Temporal bone	Glenoid Fossa	-0.080	-0.070	-0.057	-0.050	-0.053	-0.047
	Zygomatic process	-0.147	-0.331	-0.104	-0.236	-0.096	-0.221

Y = Antero-posterior displacement (+, posteriorly; −, anteriorly); Z = Vertical displacement (+, superiorly; −, inferiorly). U1 point = On the incisal edge of maxillary central incisor; L1 point = On the incisal edge of mandibular central incisor.

Interestingly, as the angulation of force application was increased from 10° to 20° then 30°, a decreasing forward displacement of maxilla was observed. Likewise, less anti-clockwise rotation of maxilla in vertical direction was observed.

### SUPERIMPOSITIONS

**Figure 10:**
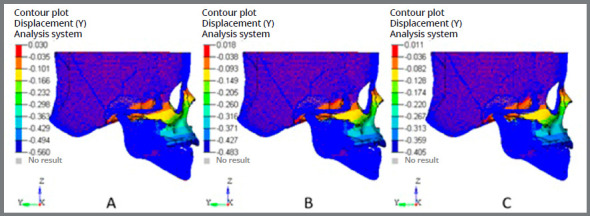
Superimposition showing antero-posterior displacement: A) 10° angulation; B) 20° angulation; C) 30° angulation.

**Figure 11:**
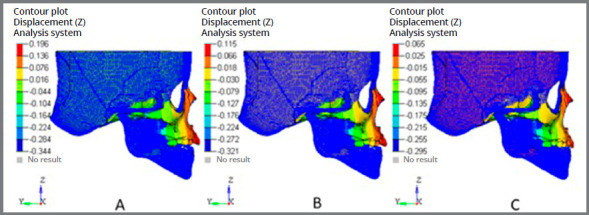
Superimposition showing vertical displacement: A) 10° angulation; B) 20° angulation; C) 30° angulation.

In the superimposition results, the ‘before’ image is shown in blue mesh, while the ‘after’ image is displayed in a range of colors that directly resemble the amount of Y- displacement (pure protraction) or Z- displacement (vertical) following force application (Figs 10 and 11).

## DISCUSSION

For correction of Class III malocclusion, studies have described an array of orthodontic and orthopedic treatments, such as: Class III functional appliances,^11^ chin guards,^12^ splints with Class III elastics,^13^ and cervical extraoral mandibular anchoring.^14^ Despite many treatment options available, the individual therapeutic objectives and the skeletal and dentoalveolar structures involved differ considerably from one technique to another. Advantages of I-SAMP include possibility of 24 hours/day intraoral traction without an external facemask, requiring less patient compliance and the non-interference of miniplates with tooth movements.^4^ ^,^ ^6^

The von Mises stress was used for this study due to the appropriateness and validity of the von Mises theory of failure.^15^ This finite element analysis study showed high von Mises stresses at the sites of miniplate placement in both maxilla and mandible, but in much less magnitude at the mandibular miniplate attachment site. A previous study by Van Hevele et al^16^ showed that the failure of miniplates was six times more frequent in the maxilla, and occurred at a faster rate in the maxilla than in the mandible. This evidence was validated by the present study, in which the mandible showed approximately one-fourth of von Mises stress when compared to the maxilla.

Mechanical stresses of appropriate strain amplitude, rate, and dose can be applied to the sutures and properly modify sutural growth.^17^ Biomechanical stresses produced by orthopedic forces are transmitted to sutures and result in anabolic changes such as widening, angiogenesis and bone apposition.^18^ In this study, it was found that the zygomaticotemporal, zygomaticomaxillary and sphenozygomatic sutures played major roles in the forward displacement and counterclockwise rotation of the maxilla, irrespective of the angulation of load application. The internasal and frontonasal sutures showed the lower amount of von Mises stresses.

The study of differential strain patterns by Oberheim and Mao^19^ showed contrasting bone-strain patterns in response to simulated orthopedic forces across zygomaticotemporal suture. Similarly, the present study also obtained differential strain patterns along the various sutures - i.e. the zygomaticomaxillary, zygomaticotemporal, and zygomaticofrontal sutures -, as they were associated with both tensile and compressive stresses. A study by Nguyen et al^20^ had found that the maxilla, the zygomas, and the maxillary incisors moved forward by one unit after the application of bone-anchored maxillary protraction. It is due to the high potential of adaptation in the zygomaticotemporal, and zygomaticofrontal sutures that displacement in both maxilla and zygomas is same (3.7mm).^21^ This study also showed displacement of zygomatic arch along with maxilla as one unit, in superimpositions (Fig 9). These findings are also supported by animal studies showing that the suture surface area and the complexity of interdigitations is higher in the zygomaticomaxillary suture than in the zygomaticotemporal and zygomaticofrontal sutures.^22^

When 250 g/side of orthopedic force was applied, it was found the maximum of traction correction force conversion into von Mises stresses in maxilla and zygomatic bone. In the present study, after loading the model with the I-SAMP, the maxilla underwent forward displacement and counterclockwise rotation, and the maxillary anterior teeth underwent labial inclination irrespective of the angulations of load application. These results were similar to clinical findings, suggesting the reasonability and feasibility of the modeling.^8^ ^,^ ^9^

If we look at the displacement values of surface landmarks of mandible, the mandible underwent negligible displacement in the form of clockwise rotation. Centre of rotation of mandible seemed to be at Gonion, as all values in all the three axes were almost zero. Previous systematic review also showed that both skeletally and dentoalveolar anchored dentofacial orthopedics resulted in the clockwise rotation of mandible and increase in lower-anterior facial height.^22^

This study has provided additional information on the effect of varying angulation of Class III elastics in case of I-SAMP, which to the best of our knowledge has not been discussed in any previous studies. The corrective effect on Class III pattern by forward displacement of zygomatico-maxillary complex, as well as rotations of the maxilla and mandible, decreased gradually with an increase of the angle of load application between miniplates from 10° to 30°. Another study^23^ concluded that determination of the traction force direction should be according to the anchorage location and skeletal characteristics of patients. The finding that the stress values of the pterygomaxillary, frontonasal, frontomaxillary, nasomaxillary and internasal sutures were higher in the 10° angulation than in the 30° (Fig 6) suggests that reducing the angulation, i.e. 10°, could transfer orthopedic forces more efficiently to these sutures.

In this study, only the initial effects of single loading were analyzed over the craniomaxillofacial complex, but not the nonlinear variations of displacement and stress occurring in long-term loading over time - a common limitation of the FEM. It should be noted that any variation in values between this study and previous studies can be attributed to the fact that we only considered forces from the intraoral Class III elastics, and not from the soft tissues such as muscles, ligaments and skin. Moreover, this finite element modeling study was performed in Freeway space state, and the upper and lower dentitions were not in contact. Hence, opposing forces from maxillary molar to mandibular molar were not deliberated.

## CLINICAL RELEVANCE

This first *in-silico* research on the I-SAMP suggests that clinicians should be aware of the fact that the prescribed angulation of Class III elastics should be as low as possible, since the displacement of zygomatico-maxillary complex and mandible increases with the decrease in the angulation of elastics.

## CONCLUSIONS

Within the limitations of this study, the following conclusions were drawn:

1. The magnitude of von Mises stresses on the craniofacial sutures with this treatment modality was in the range of 0.002 to 0.044 MPa.
2. The zygomaticotemporal, zygomaticomaxillary, and sphenozygomatic sutures played major roles in the forward displacement and counterclockwise rotation of the maxilla, irrespective of the angulation of load application. The internasal and frontonasal sutures showed the lower amount of von Mises stresses.
3. Conversely, the mandible showed one-fourth of von Mises stresses and negligible displacement with clockwise rotation, when compared to the maxilla.
4. The displacements, as well as rotations of the craniomaxillary complex and mandible, decreased gradually with an increase of the angle of load application between miniplates from 10° to 30°.
5. The treatment changes in both upper and lower incisors followed the observed changes in the maxilla and mandible, respectively.
